# Supplementary material for: Array detection enables large localization range for simple and robust MINFLUX
Source: Light Sci Appl. 2025 Jul 3;14:234. doi: 10.1038/s41377-025-01883-1 (PMC12229606; doi:10.1038/s41377-025-01883-1)
Supplement: Supplementary file 1 — Supplemental Material [file 41377_2025_1883_MOESM1_ESM.pdf]

Supplementary Information

for

Array Detection Enables Large Localization Range for Simple and Robust MINFLUX

Eli Slenders, Sanket Patil, Marcus Oliver Held, Alessandro Zunino, and Giuseppe Vicidomini

Supplementary Note 1: ISM-FLUX theory

Consider a system in which a fluorophore, i.e., a point-emitter, is sequentially illuminated with K illumination patterns, e.g. by moving a doughnut beam to different positions. The number of photons detected with a single-element detector can be assumed to be Poisson distributed with λ_i the expected number of photons under illumination i ($i \in \{1,2,...,K\}$). Thus, the probability of observing $\mathbf{n} = (n_1, n_2, ..., n_K)$ photons is

$$P(\mathbf{n}|\{\lambda_i\}) = \prod_{i=1}^{K} \frac{e^{-\lambda_i}\lambda^{n_i}}{n_i!} \tag{S1}$$

However, this is the probability of having $\mathbf{n}$ photons. What we are interested in is the probability $P(\mathbf{n}|N)$, with $N = \sum_i n_i$. Using Bayes' theorem, we have

$$P(\mathbf{n}|N,\{\lambda_i\}) = \frac{P(\mathbf{n}|\{\lambda_i\})}{P(N|\{\lambda_i\})} = \frac{\prod_{i=1}^{K} \frac{e^{-\lambda_i}\lambda^{n_i}}{n_i!}}{\frac{e^{-\lambda_{tot}}\lambda_{tot}^{N}}{N!}} = \frac{N!\prod_{i=1}^{K} \frac{e^{-\lambda_i}\lambda^{n_i}}{n_i!}}{e^{-\lambda_{tot}}\lambda_{tot}^{N}} \tag{S2}$$

with $\lambda_{tot} = \sum \lambda_i$.

We can write $\lambda_i = Np_i$, with N the total number of expected detected photons, p_i the probability that if a photon is detected, it is detected under illumination i, and $\sum_i p_i = 1$. Then,

$$P(\mathbf{n}|N,\{p_i\}) = \frac{N!\prod_{i=1}^{K}\left(\frac{e^{-Np_i}N^{n_i}p_i^{n_i}}{n_i!}\right)}{e^{-N}N^{N}} = \frac{N!}{\prod_{i=1}^{K} n_i!}\prod_{i=1}^{K} p_i^{n_i} \tag{S3}$$

which is a multinomial distribution.

We now turn to the ISM-FLUX case with an array detector. For ISM-FLUX, we can define a probability p_{ij} that if a photon detection event happens, it happens under illumination i in detector-element j. We can write the dependence of $p_{ij}(\mathbf{r}_E)$ on the emitter position $\mathbf{r}_E$. Assume a (doughnut) illumination intensity profile $h(\mathbf{r}-\mathbf{r}_i)$ that moves to K different positions $\mathbf{r}_i$. Assume further that the expected number of emitted photons is proportional to the illumination intensity. Then, the fraction of signal photons emitted under illumination i is:

$$f_i(\mathbf{r}_E) = \frac{h(\mathbf{r}_E - \mathbf{r}_i)}{\sum_{p=1}^{K} h(\mathbf{r}_E - \mathbf{r}_p)} \tag{S4}$$

The fluorescence emitted by the single emitter follows a spatial distribution $h_2(\mathbf{r}-\mathbf{r}_E)$ equal to the emission PSF centered at position $\mathbf{r}_E$. We have,

$$\int_{\mathbb{R}^2} h_2(\mathbf{r}-\mathbf{r}_E)\,dx\,dy = 1 \tag{S5}$$

The probability $g_{ij}(\mathbf{r}_E)$ that a photon emitted under illumination i is detected by detector element j is

$$g_{ij}(\mathbf{r}_E) = \int_{\mathbb{R}^2} h_2(\mathbf{r}-\mathbf{r}_E)w_{ij}(\mathbf{r})\,dx\,dy \tag{S6}$$

Here, $w(\mathbf{r})$ is a 2D window function describing the detector element. Note that, since the detector is placed in descanned mode, the detector *moves* when the excitation beam moves, hence the dependence on both the indices i and j:

$$w_{ij}(\mathbf{r}) = w(\mathbf{r}-\mathbf{r}_i-\mathbf{s}_j) \tag{S7}$$

with $\mathbf{s}_j$ the position of detector element j within the array. Note that the detector has a finite number of elements and thus a finite size hence, not all emitted photons will be detected:

$$\sum_j g_{ij}(\mathbf{r}_E) = \sum_j \int_{\mathbb{R}^2} h_2(\mathbf{r}-\mathbf{r}_E)w_{ij}(\mathbf{r})\,dx\,dy = \int_{\mathbb{R}^2} h_2(\mathbf{r}-\mathbf{r}_E)\sum_j w_{ij}(\mathbf{r})\,dx\,dy = \int_A h_2(\mathbf{r}-\mathbf{r}_E)\,dx\,dy < 1 \tag{S8}$$

with A the overall area of all detector elements.

If a photon is observed, the probability it happened under illumination i in detector element j is:

$$p_{ij} = \frac{P(\text{detection event in i,j})}{P(\text{detection event})} = \frac{f_i(\mathbf{r}_E)g_{ij}(\mathbf{r}_E)}{\sum_{p=1}^{K}\sum_{q=1}^{L} f_p(\mathbf{r}_E)g_{pq}(\mathbf{r}_E)} \tag{S9}$$

Plugging in S4 yields

$$p_{ij} = \frac{\frac{h(\mathbf{r}_E-\mathbf{r}_i)}{\sum_{p=1}^{K} h(\mathbf{r}_E-\mathbf{r}_p)} g_{ij}(\mathbf{r}_E)}{\sum_{p=1}^{K}\left(\frac{h(\mathbf{r}_E-\mathbf{r}_p)}{\sum_{q=1}^{K} h(\mathbf{r}_E-\mathbf{r}_q)} \sum_{q=1}^{L} g_{pq}(\mathbf{r}_E)\right)} = \frac{h(\mathbf{r}_E-\mathbf{r}_i)g_{ij}(\mathbf{r}_E)}{\sum_{p=1}^{K}\sum_{q=1}^{L} h(\mathbf{r}_E-\mathbf{r}_p)g_{pq}(\mathbf{r}_E)} \tag{S10}$$

where, using Eq. S6, the numerator can be written as

$$I_{ij}(\mathbf{r}_E) = h(\mathbf{r}_E-\mathbf{r}_i)\int_{\mathbb{R}^2} h_2(\mathbf{r}-\mathbf{r}_E)w(\mathbf{r}-\mathbf{r}_i-\mathbf{s}_j)\,dx\,dy = h(\mathbf{r}_E-\mathbf{r}_i)\int_{\mathbb{R}^2} h_2(\mathbf{r})w(\mathbf{r}-\mathbf{s}_j+\mathbf{r}_E-\mathbf{r}_i)\,dx\,dy \tag{S11}$$

For $\mathbf{r}_i = 0$, we find the equation for the signal in detector element j for a point-source at position $\mathbf{r}_E$ and detector element at position $\mathbf{s}_j$. Experimentally, one can measure $I_{0j}(\mathbf{r}_E)$ by moving a point source in 2D with a piezoelectric stage and measuring the resulting signal for each position and in each detector element or, equivalently, by scanning the point source with the galvanometric scanners.

For $\mathbf{r}_i \neq 0$, we have $I_{ij}(\mathbf{r}_E) = I_{0j}(\mathbf{r}_E - \mathbf{r}_i)$. Thus, when the TCP is known, one can simply shift the resulting point-source images to the right positions to get the full set of $I_{ij}(\mathbf{r}_E)$. One can find the denominator in Eq. S10 by summing all images. For the ISM-FLUX setup in this work, we have $K = 25$ detector elements and $L = 32$ positions.

In the presence of background, Eq. S10 becomes:

$$p_{ij} = \frac{h(\mathbf{r}_E-\mathbf{r}_i)g_{ij}(\mathbf{r}_E)+b_{ij}}{\sum_{p=1}^{K}\sum_{q=1}^{L}\left(h(\mathbf{r}_E-\mathbf{r}_p)g_{pq}(\mathbf{r}_E)+b_{pq}\right)} \tag{S12}$$

with b_{ij} the probability that a count is a background count in element j under illumination i. With a SPAD array detector, the main contributions to the background counts are the out-of-focus fluorescence and the dark counts. We assume that both contributions are equal for each detector element and each illumination, thus $b_{ij} = b$:

$$p_{ij} = \frac{h(\mathbf{r}_E-\mathbf{r}_i)g_{ij}(\mathbf{r}_E)+b}{KLb+\sum_{p=1}^{K}\sum_{q=1}^{L} h(\mathbf{r}_E-\mathbf{r}_p)g_{pq}(\mathbf{r}_E)} \tag{S13}$$

Define the signal-to-background ratio as:

$$\mathrm{SBR}(\mathbf{r}_E) = \frac{\sum_{p=1}^{K}\sum_{q=1}^{L} h(\mathbf{r}_E-\mathbf{r}_p)g_{pq}(\mathbf{r}_E)}{KLb} = \frac{\sum_p\sum_q h_p(\mathbf{r}_E)g_{pq}(\mathbf{r}_E)}{KLb} \tag{S14}$$

Where we introduced a shorter notation for the double sum. Then,

$$p_{ij}(\mathbf{r}_E) = \frac{h(\mathbf{r}_E-\mathbf{r}_i)g_{ij}(\mathbf{r}_E)+b}{KLb+KLb\,\mathrm{SBR}(\mathbf{r}_E)} \tag{S15}$$

$$= \frac{h(\mathbf{r}_E-\mathbf{r}_i)g_{ij}(\mathbf{r}_E)\sum_p\sum_q h_p(\mathbf{r}_E)g_{pq}(\mathbf{r}_E)}{KLb(\mathrm{SBR}(\mathbf{r}_E)+1)\sum_p\sum_q h_p(\mathbf{r}_E)g_{pq}(\mathbf{r}_E)} + \frac{b}{KLb(\mathrm{SBR}(\mathbf{r}_E)+1)} \tag{S16}$$

$$= \frac{h(\mathbf{r}_E-\mathbf{r}_i)g_{ij}(\mathbf{r}_E)\mathrm{SBR}(\mathbf{r}_E)}{(\mathrm{SBR}(\mathbf{r}_E)+1)\sum_p\sum_q h_p(\mathbf{r}_E)g_{pq}(\mathbf{r}_E)} + \frac{b}{KLb(\mathrm{SBR}(\mathbf{r}_E)+1)} \tag{S17}$$

$$= \frac{\mathrm{SBR}(\mathbf{r}_E)}{\mathrm{SBR}(\mathbf{r}_E)+1}\frac{h(\mathbf{r}_E-\mathbf{r}_i)g_{ij}(\mathbf{r}_E)}{\sum_p\sum_q h_p(\mathbf{r}_E)g_{pq}(\mathbf{r}_E)} + \frac{1}{\mathrm{SBR}(\mathbf{r}_E)+1}\frac{1}{KL} \tag{S18}$$

Assuming no prior information, we can express a likelihood function $\mathcal{L}$ as

$$\mathcal{L}(\mathbf{r}_E|\{n_{ij}\}) = \frac{N!}{\prod_{i=1}^{K}\prod_{j=1}^{L} n_{ij}!}\prod_{i=1}^{K}\prod_{j=1}^{L} p_{ij}(\mathbf{r}_E)^{n_{ij}} \tag{S19}$$

with p_{ij} from Eq. S18.

The log-likelihood function, dropping constant terms, is

$$\ell(\mathbf{r}_E|\{n_{ij}\}) = \sum_{i=1}^{K}\sum_{j=1}^{L} n_{ij}\ln p_{ij}(\mathbf{r}_E) \tag{S20}$$

The maximum likelihood estimation for the emitter position is

$$\mathbf{r}_E^{\mathrm{MLE}} = \arg\max(\ell(\mathbf{r}_E|\{n_{ij}\})) \tag{S21}$$

Supplementary Note 2: Bias of the maximum-likelihood estimator in ISM-FLUX

The maximum-likelihood estimator (MLE) is not inherently unbiased. Especially in the case of small samples, i.e. low photon counts, there may be a difference between the expected value of the emitter position and the ground truth value. To understand the bias of the MLE for ISM-FLUX, we simulated ISM-FLUX measurements for different emitter positions and different photon counts and compared the position retrieved from the MLE with the ground truth. We consecutively positioned the emitter at various points along a line passing through the TCP center. We used the simulated MDFs, Fig. S12, to draw photon counts from a multinomial distribution with relative probabilities equal to the MDF values at the emitter position. We assumed 0 background. Then, we used the MLE to retrieve the emitter position and stored the difference in (x, y) between the ground truth and the retrieved position. We repeated this simulation 1000$\times$ for each emitter position and each photon count number.

The result, Fig. S1, shows that the MLE is always unbiased in the TCP center and on the TCP circle, regardless of the number of photon counts. For other positions within the TCP, as well as outside the TCP, we find a position- and photon-count-dependent bias. For high photon counts, i.e. $N > 1000$, the bias is far below 1 nm in both directions for all positions within the detector FOV and the MLE can be considered unbiased. For lower photon counts, the bias increases in both directions but remains below 10 nm for the whole FOV for $N \geq 30$. Note that for most positions on the horizontal line, the bias in the x (radial) direction is higher than in the y (tangential) direction.

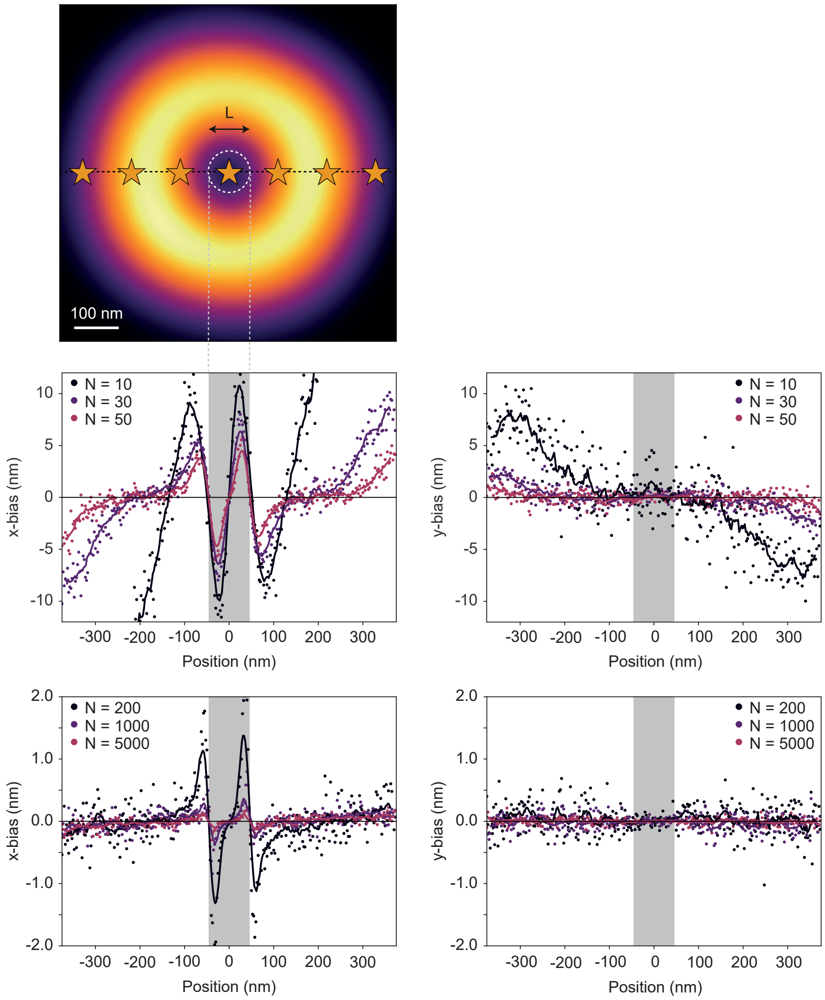

Fig. S1. Bias of the MLE in the x and y directions for different photon counts (N) and various emitter positions. $L = 90$ nm. The emitter is moved to 300 positions in 2.5 nm steps along a horizontal line passing through the TCP center (indicated by the dotted black line in the intensity plot). For each position, the bias in both directions is plotted. The scatter plots display the raw data, while the line plots show the corresponding moving averages with a window size of 10.

Supplementary Note 3: Theory of ISM-FLUX self-calibration

Consider a molecule at position $\mathbf{r}_E$ and two opposite points on the TCP, located at $\mathbf{r}_1$ and $\mathbf{r}_{16}$. When the laser beam is at position $\mathbf{r}_1$ or $\mathbf{r}_{16}$, the detector center is also at $\mathbf{r}_1$ or $\mathbf{r}_{16}$, respectively. Thus, in the detector frame-of-reference and assuming no (shot) noise, the image of the molecule moves from being centered around $\mathbf{r}_E - \mathbf{r}_1$ to $\mathbf{r}_E - \mathbf{r}_{16}$. The shift of the molecule $\mathbf{L}$ as seen by the detector is:

$$\mathbf{L} = \mathbf{r}_{16} - \mathbf{r}_1, \tag{S22}$$

with $|\mathbf{L}| = L$, the diameter of the TCP. Thus, L can be directly derived from two micro-images taken at opposite angles of the TCP.

However, $\mathbf{r}_1$ and $\mathbf{r}_{16}$ are experimental localizations subject to a non-zero localization uncertainty. As a more robust approach, we propose to localize events for different points on the TCP, assuming a TCP diameter ℓ. In other words, we shift the two emitter positions in the detector reference frame with $\pm\boldsymbol{\ell}/2$, with $\boldsymbol{\ell}$ a vector on the line connecting the two points on the TCP and $|\boldsymbol{\ell}| = \ell$:

$$\boldsymbol{\ell} = \ell \frac{\mathbf{r}_{16} - \mathbf{r}_1}{|\mathbf{r}_{16} - \mathbf{r}_1|} \tag{S23}$$

The two emitter positions are then

$$\mathbf{r}_E - \mathbf{r}_1 - \boldsymbol{\ell}/2, \tag{S24}$$

$$\mathbf{r}_E - \mathbf{r}_{16} + \boldsymbol{\ell}/2. \tag{S25}$$

The difference $\Delta\mathbf{r}$ between the two positions is

$$\Delta\mathbf{r} = -\mathbf{r}_1 + \mathbf{r}_{16} - \boldsymbol{\ell} = \mathbf{L} - \boldsymbol{\ell} \tag{S26}$$

For $\boldsymbol{\ell} = \mathbf{L}$, $\Delta\mathbf{r} = 0$.

In reality, $\mathbf{r}_1$ and $\mathbf{r}_{16}$ are localizations with uncertainty σ^2. Assuming the localizations can be drawn from a Gaussian distribution with standard deviation σ, the mean squared distance $\langle d^2 \rangle$ between the two positions and their mean is

$$\langle d^2 \rangle = \left(\frac{|\Delta\mathbf{r}|}{2} \right)^2 + \sigma^2 = \frac{(L-\ell)^2}{4} + \sigma^2 \tag{S27}$$

Thus, $\langle d^2 \rangle$ reaches a minimum where $\ell = L$, and the function value at this point corresponds to the experimental localization uncertainty.

Since the photon counts in a single-molecule on-event are rather low, we split the data from each event into 4 circle segments of 8 consecutive points in the orbit. Then, we approximate $\langle d^2 \rangle$ as the mean squared displacement between the four localizations and their mean. As a metric for the overall localization spread of all events combined, we take the median value of all $\langle d^2 \rangle$ values. We repeat this calculation for a set of ℓ values and we fit the resulting curve with a second-order polynomial. The ℓ value that minimizes the localization spread corresponds to the true L value. The protocol is illustrated in Fig. S10.

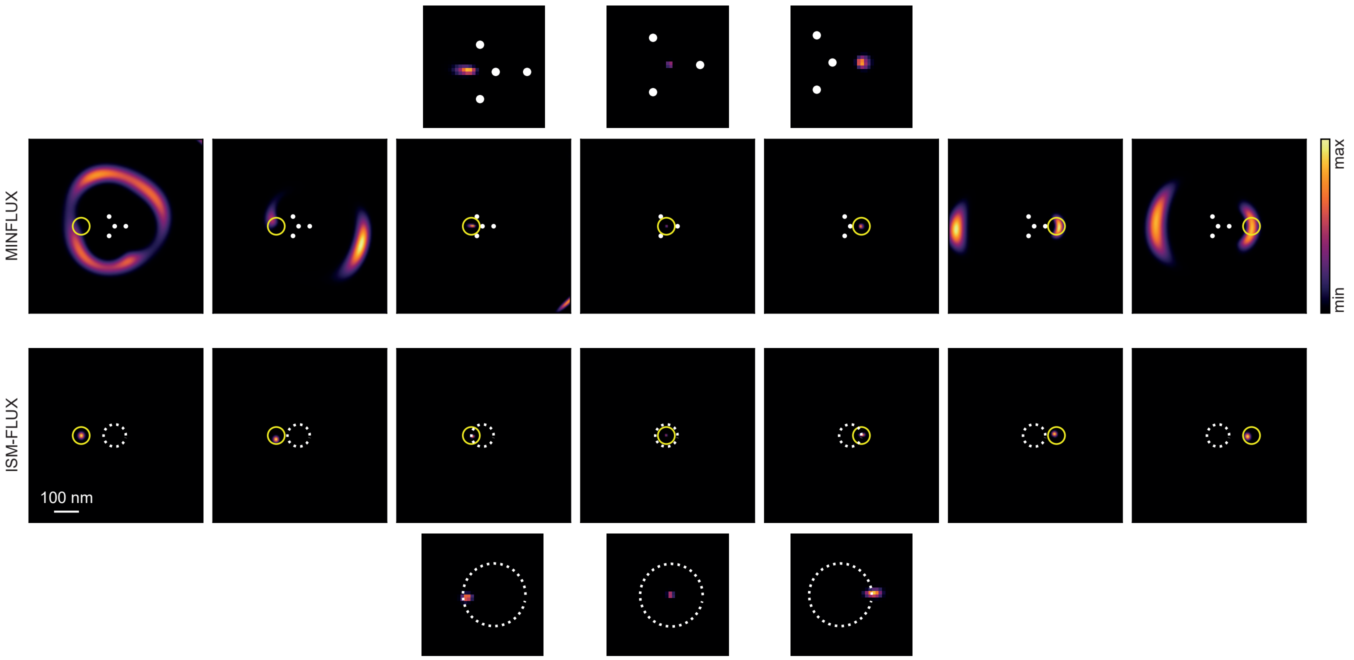

Fig. S2. Likelihood maps for simulated MINFLUX and ISM-FLUX measurements. For MINFLUX, we considered a TCP, indicated in white, consisting of 3 evenly spaced points on a circle with a diameter L = 90 nm and one point in the center of this circle. For ISM-FLUX, the TCP consists of 32 points on a circle with a diameter of 90 nm, without the central point. We placed the emitter at 7 different positions in steps of 45 nm, indicated by the yellow circles, and we calculated the MLE maps for $N = 200$ photons. For visualization purposes, some TCP points are not shown. The top and bottom row images show zoomed-in MLE maps of the three emitter positions closest to the TCP center.

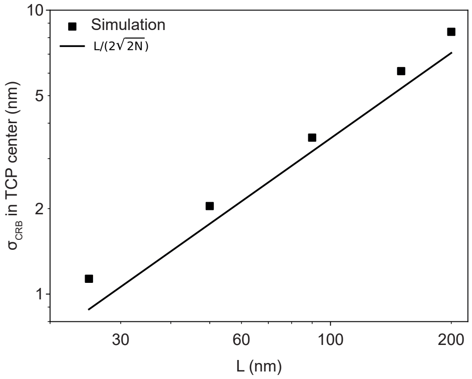

Fig. S3. Simulated σ_{CRB} in the TCP center as a function of L for 100 photons. The full line corresponds to the theoretical MINFLUX localization uncertainty for 100 photons.

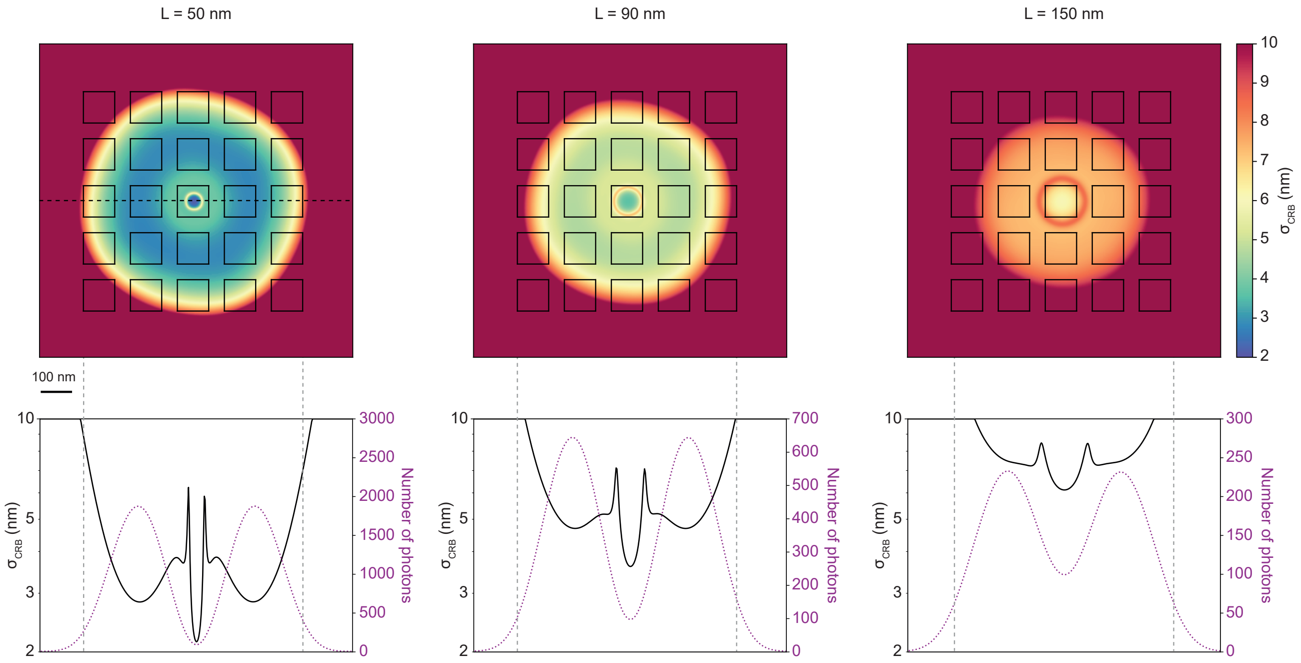

Fig. S4. CRB for a molecule emitting $N = 100$ photons in the TCP center. N is rescaled for the other emitter positions, assuming $N(\mathbf{r})$ is proportional with $\sum_{i=1}^{32}\sum_{j=1}^{25} p_{i,j}(\mathbf{r})$.

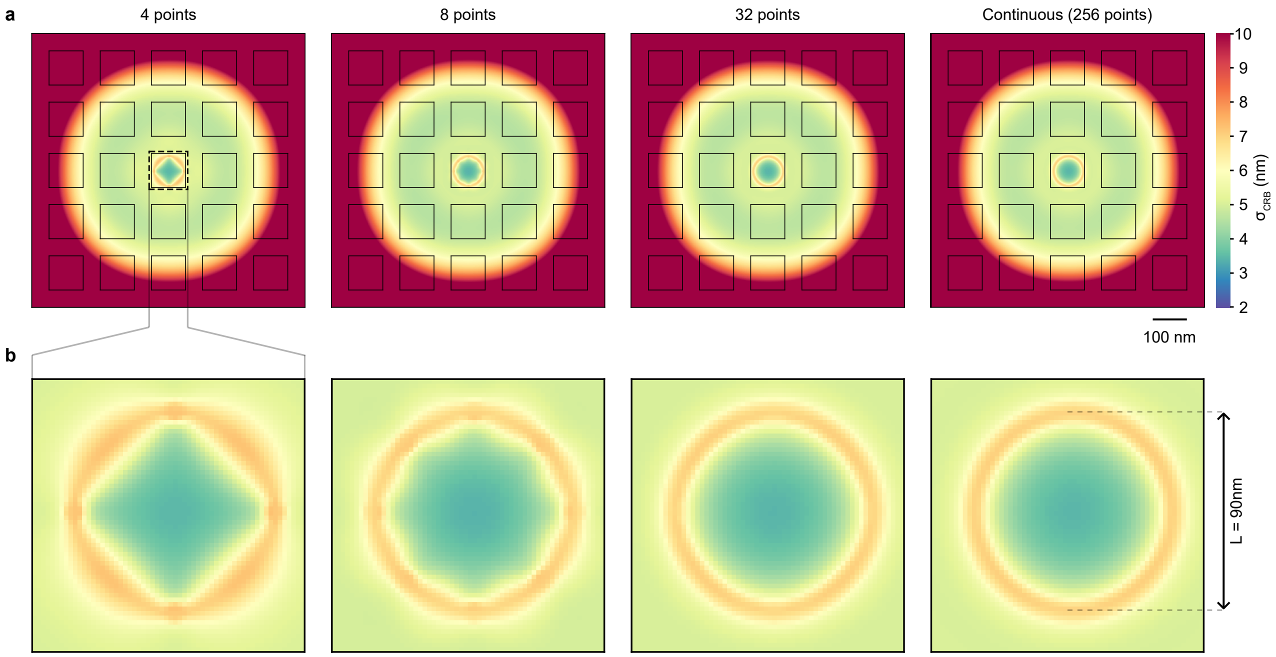

Fig. S5. (a) CRB for a molecule emitting $N = 100$ photons in the TCP center for different number of points during the orbital scanning. N is rescaled for the other emitter positions. $L = 90$ nm. (b) Zoomed-in section of the center of the CRB map.

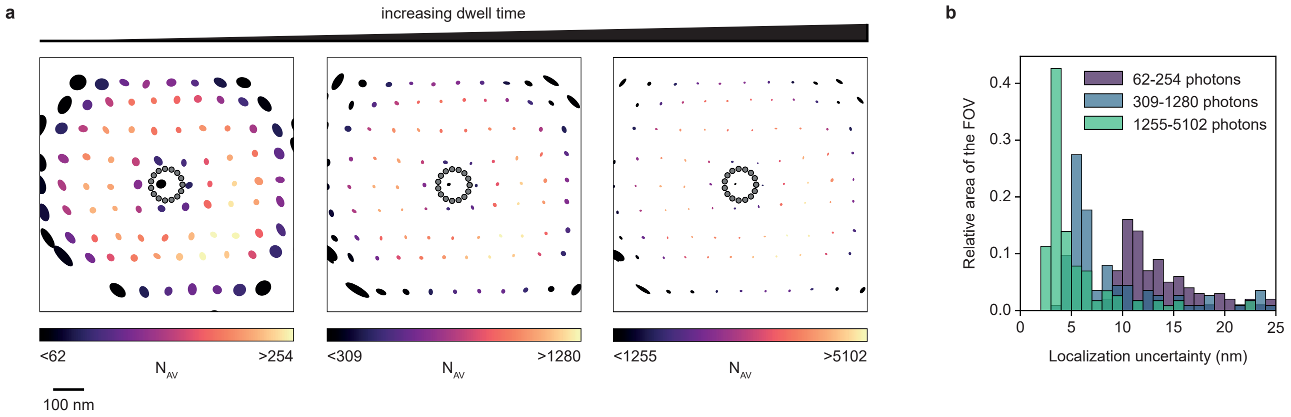

Fig. S6. (a) Calculated localization uncertainty over a large FOV, obtained by moving the GNP to 170 positions. The color shows the number of detected photon counts for a constant dwell time. The minimum color values (i.e., 62, 309, 1255) correspond to the photon counts for the TCP center. Close to the FOV edge, the actual number of photons may be lower, as indicated by the <-sign in the color bar. The size of the ellipses represents the localization uncertainty along two axes, see Methods. (b) Histograms of the data from (a), in which the localization uncertainty is defined as the average of half of the width and height of the ellipses. The relative area of the FOV is approximated as the number of positions in (b) divided by the total number of positions to which we moved the nanoparticle.

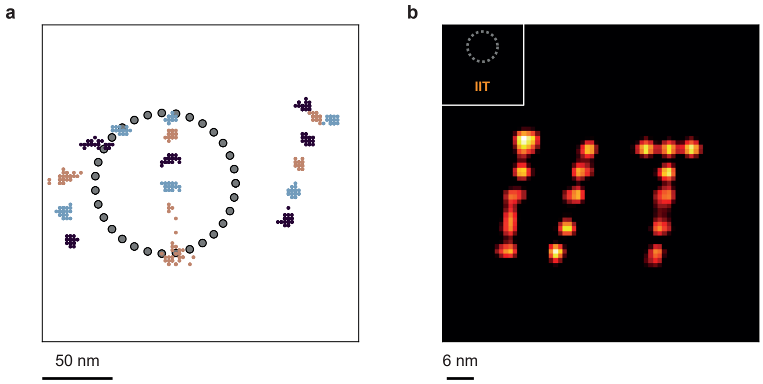

Fig. S7. GNP results for simulated MDFs. For (b), the GNP from the time was outside the TCP, as indicated in the inset.

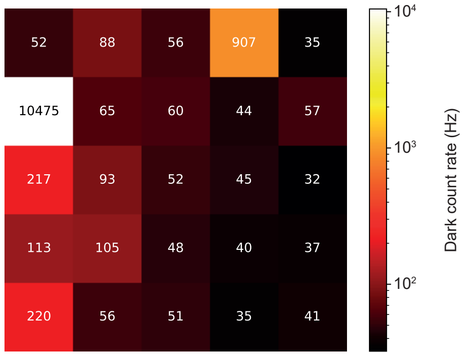

Fig. S8. Dark count rate for the 25 most central detector elements of the 7x7 array. Pixel 3 (fourth pixel of the first row), and pixel 5 (first pixel of the second row) are hot elements and were – unless mentioned otherwise – excluded from the data analysis. Dark count rates for each channel were obtained by covering the detector and fitting to a line the photon counts accumulated over a two-second period.

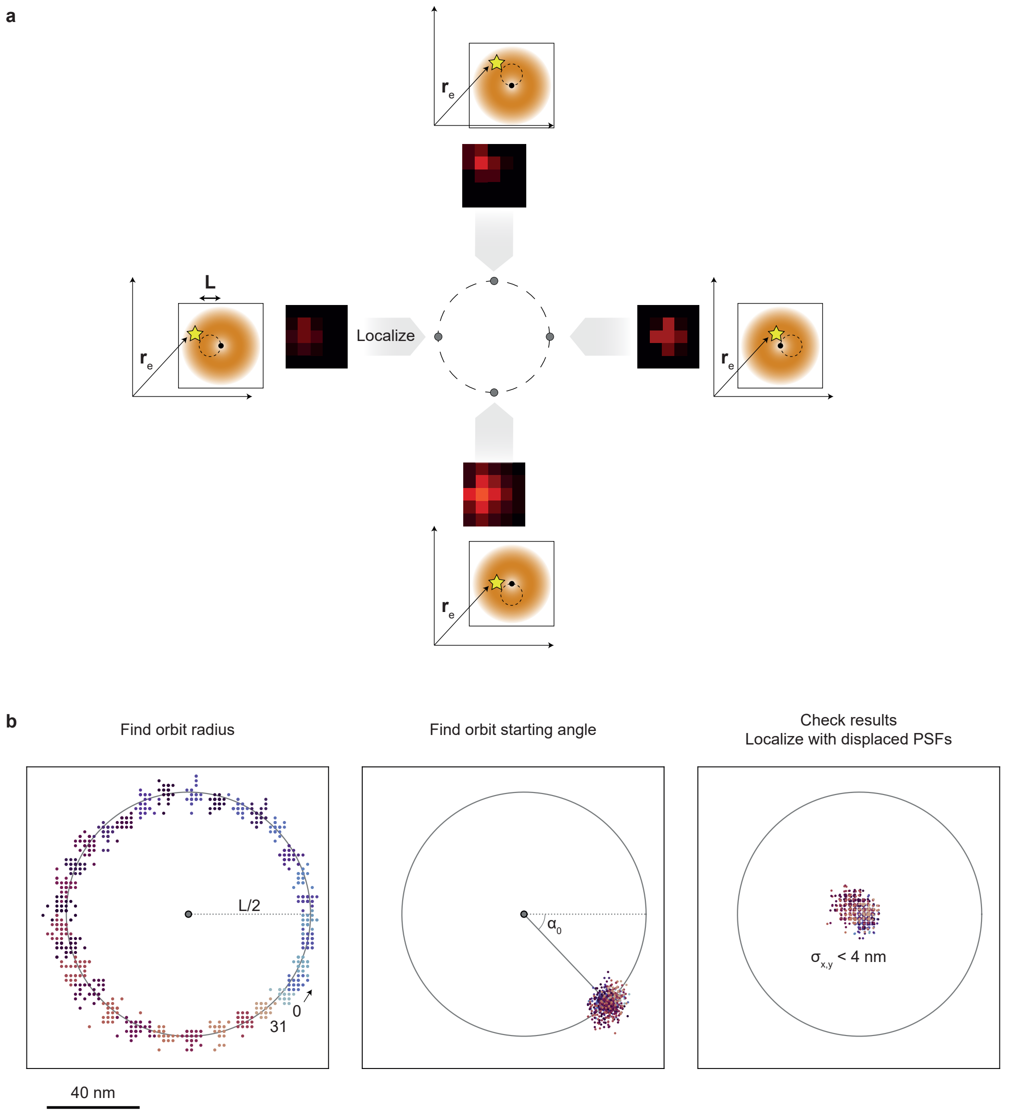

Fig. S9. Measurement of the circular orbit parameters from a reference measurement. (a) A fiducial marker is imaged while scanning the sample with a circular motion. Each position on the circle leads to a different micro-image, both in terms of the center of mass as well as the number of photons. The marker is localized in each micro-image individually using the 5x5 PSFs. Note that in this case only the distribution of the scattered photons on the detector is used, not the intensity differences between different images. Hence, also a Gaussian beam can be used for calibrating the galvanometric scanners. (b) Analyzing the 32 micro-images using an MLE leads to a distribution of localizations on a circle from which the center is estimated by taking the mean coordinates and the radius is estimated as the mean distance from the center. Mean and standard deviation over 30 circles yields $L = (105.2 \pm 0.9)$ nm. Next, all points are rotated back an integer number of $1/32 * 2\pi$ and from the mean resulting coordinates, the starting angle of the orbit is estimated. Having both the orbit radius and starting angle, a new series of PSFs can be calculated: one set of 25 PSFs for each of the 32 positions on the circle, calculated by displacing each set of 25 PSFs according to its position in the orbit. The resulting set of 32x25 PSFs is used to analyze ISM-FLUX data, i.e. data from a full orbit or a sum over several consecutive orbits will result in a single localization, in which both the distributions of the photons on the detector as well as the change in photon flux during the orbit is taken into account.

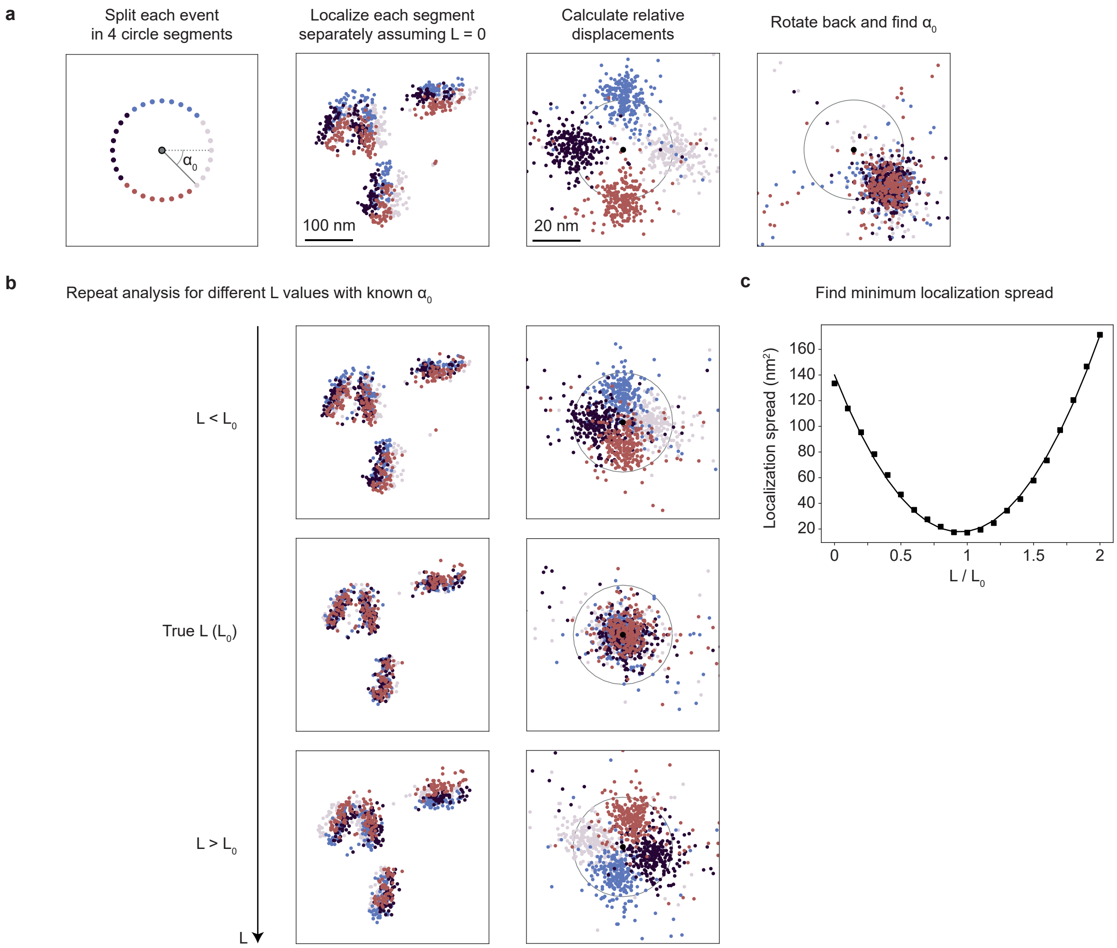

Fig. S10. ISM-FLUX is self-calibrating: measurement of the orbit parameters from the SM data. (a) Extracting the starting angle of the orbit α_0. Each blinking event is split into four segments, indicated by the different colors. The MLE is applied to each segment separately assuming $L = 0$. The result is four sets of segment-localizations that are displaced with respect to each other. Calculating for each event the relative displacement between the segments (i.e., the displacement between the localization of each segment and the mean of the four segments) results in four clusters on a circle. By rotating each segment s back by an amount equal to $-\pi/4 - s\pi/2$ with $s \in \{0, 1, 2, 3\}$, all clusters overlap and the starting angle α_0 can be measured. We calculated the median (x,y) position of each cluster and estimated α_0 as the mean $\pm$ standard deviation, which is $\alpha_0 = -0.81 \pm 0.02$, in good agreement with the value of $\alpha_0 = -0.812 \pm 0.008$ found in a reference measurement, Fig. S9. (b) The orbit diameter is found by repeating the analysis assuming different L values. For each L value, the localization spread, as defined in Sup. Note 3, is calculated from the relative displacements. (c) The localization spread as a function of L (scatter plot) is fitted with a second-order polynomial curve (line plot). The fit, $y = 137x^2 - 259x + 140$ has a minimum around $L/L_0 = 0.95$, with $L_0 = 61.4$ nm, the diameter found in the reference measurement.

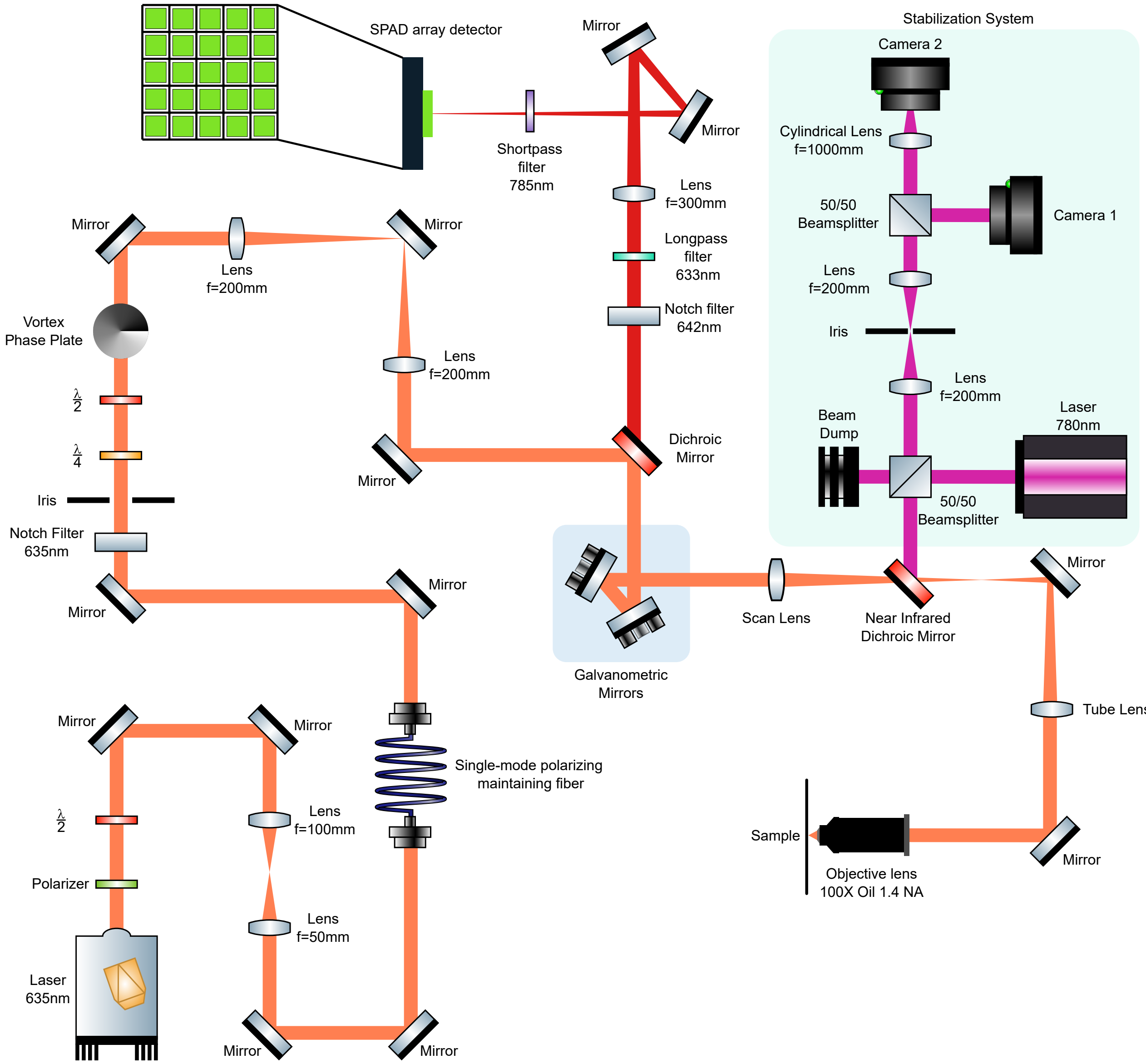

Fig. S11. Detailed optical setup. $\frac{\lambda}{2}$ and $\frac{\lambda}{4}$ stands for half-wave and quarter-wave plate. Near infrared dichroic mirror used in this system is Shortpass 750nm. The optical setup highlighted in green is the stabilization system used to keep the sample in focus during the imaging session.

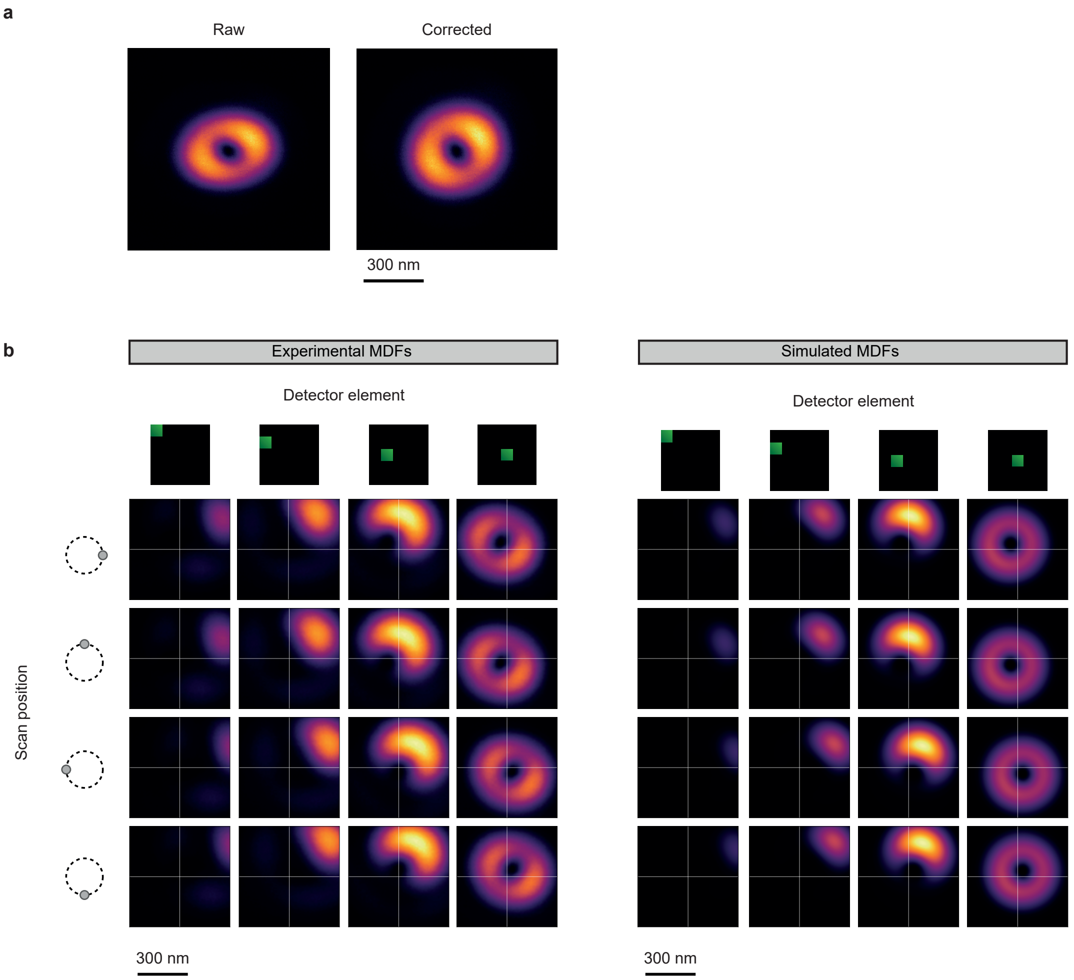

Fig. S12. (a) Experimental MDF for the central detector element, obtained by scanning a gold nanoparticle. Scan settings: 2000 $\times$ 2000 px, 2 nm/px, px dwell time 30 μs, 10 repetitions. Galvanometric limitations lead to an apparent astigmatic image, corrected in post-processing. (b) Subset of (a) experimental and (b) simulated MDFs. The full MDF data set is a collection 25 x 32 images, a combination of 25 detector elements and 32 scan positions. For simplicity, we show 4 positions and 4 detector elements. The experimental MDFs are stretch-corrected and smoothed in post-processing.

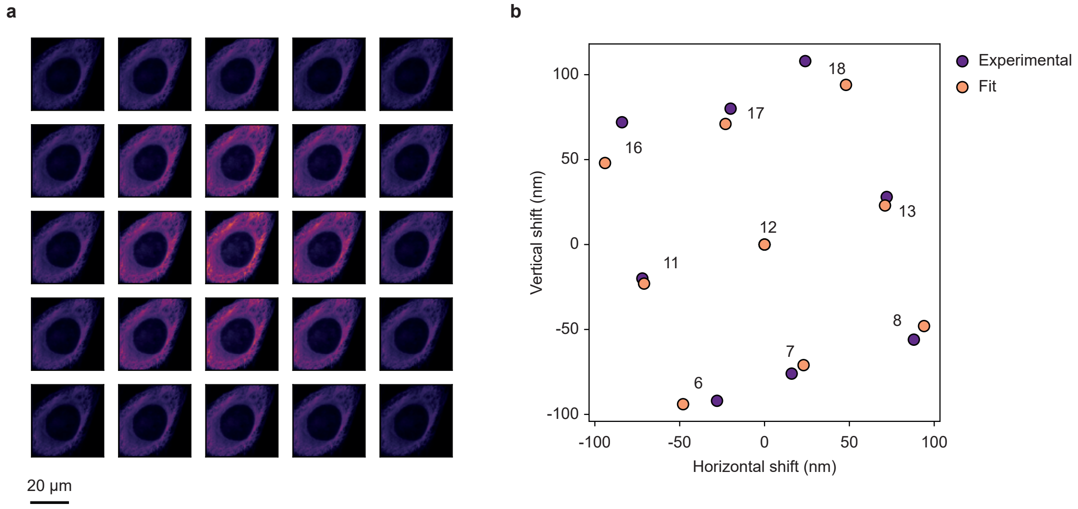

Fig. S13. Extracting the detector orientation, rotation, and system magnification from the shift vectors of an ISM data set. (a) Image of a fixed HeLa cell with α-tubulin staining. The images of the 5x5 detector elements are shown. (b) From the shift vectors of the 3x3 detector elements closest to the detector center, the detector orientation, rotation, and system magnification were estimated.

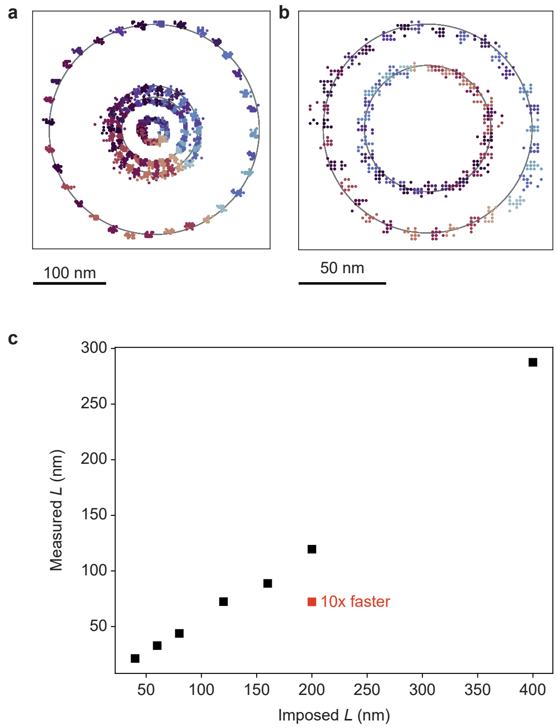

Fig. S14. (a) 32 positions of the TCP measured for different L values. From smallest to largest TCP: L = 60 nm, 160 nm, 200 nm, 400 nm. Orbit direction counterclockwise, from blue to red. (b) The same imposed L of 100 nm results in a different observed L and starting angle, depending on the scan speed. The smaller circle corresponds to a 10x faster scan (192 μs per circle) than the larger circle (c) Imposed L vs. measured L. Except for the indicated data point, all orbit times were 1.92 ms per circle.

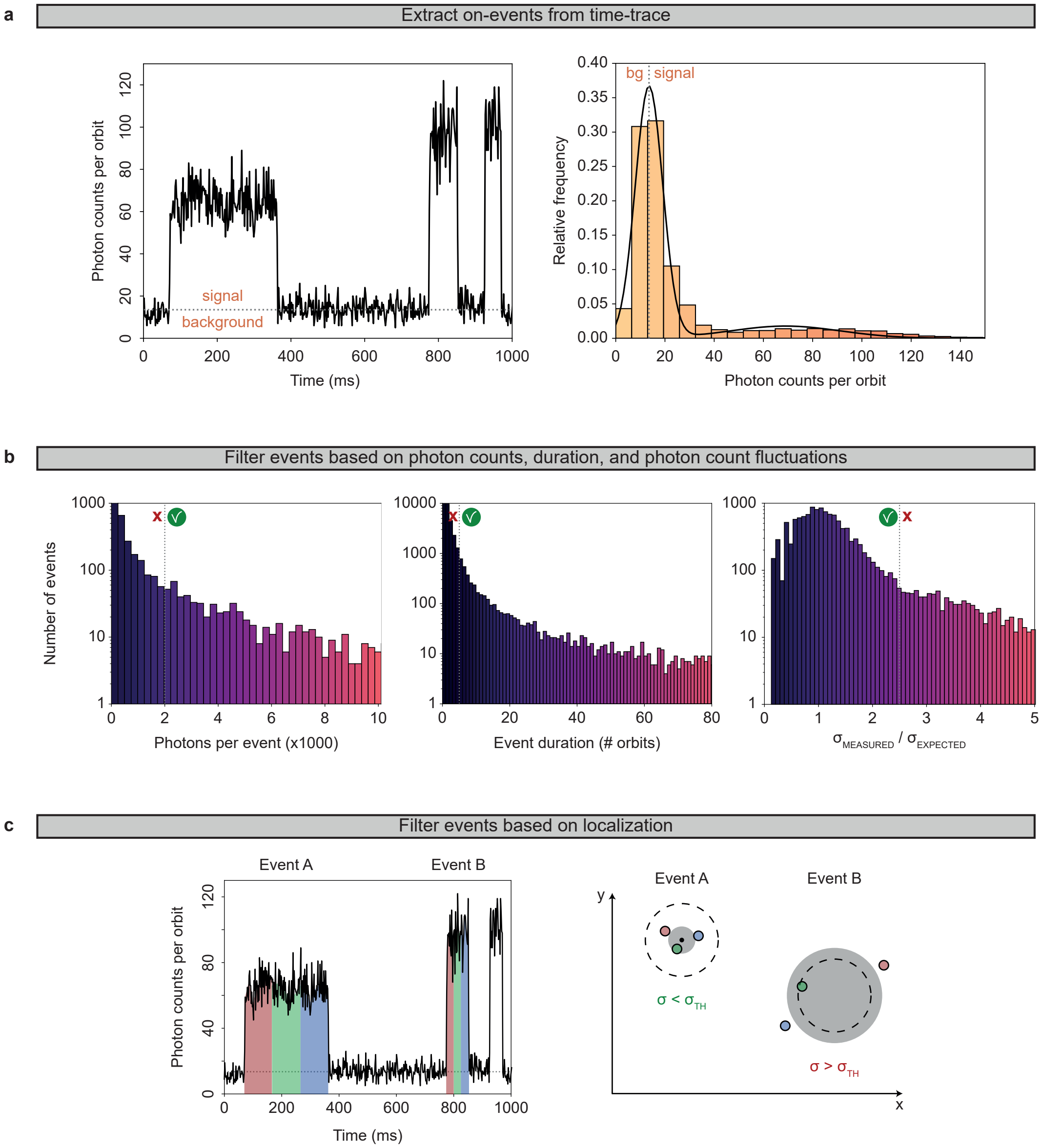

Fig. S15. Analysis protocol for ISM-FLUX on DNA-origami measured with DNA-PAINT. (a) Part of the time trace, obtained by summing all photons in all detector channels for all positions on an orbit, and histogram and fit of the full time trace (bg = background). (b) All events pass through three filters, based on the total number of photons (left), the duration (center), and the standard deviation σ_{measured} of the count fluctuations within an event (right). (c) Each event that passes all filters is split into three equally long chunks, resulting in three independent localizations, colored in red, green, and blue. If the three localizations are closer to each other than a user-chosen threshold, i.e. $\sigma < \sigma_{TH}$, the event is accepted, otherwise, the event is discarded. The grey circle has a radius of σ, the dotted line indicates the threshold. Here, event A is accepted, and the three localizations are merged into a single one, obtained by taking the mean coordinates (black dot). Event B is discarded.

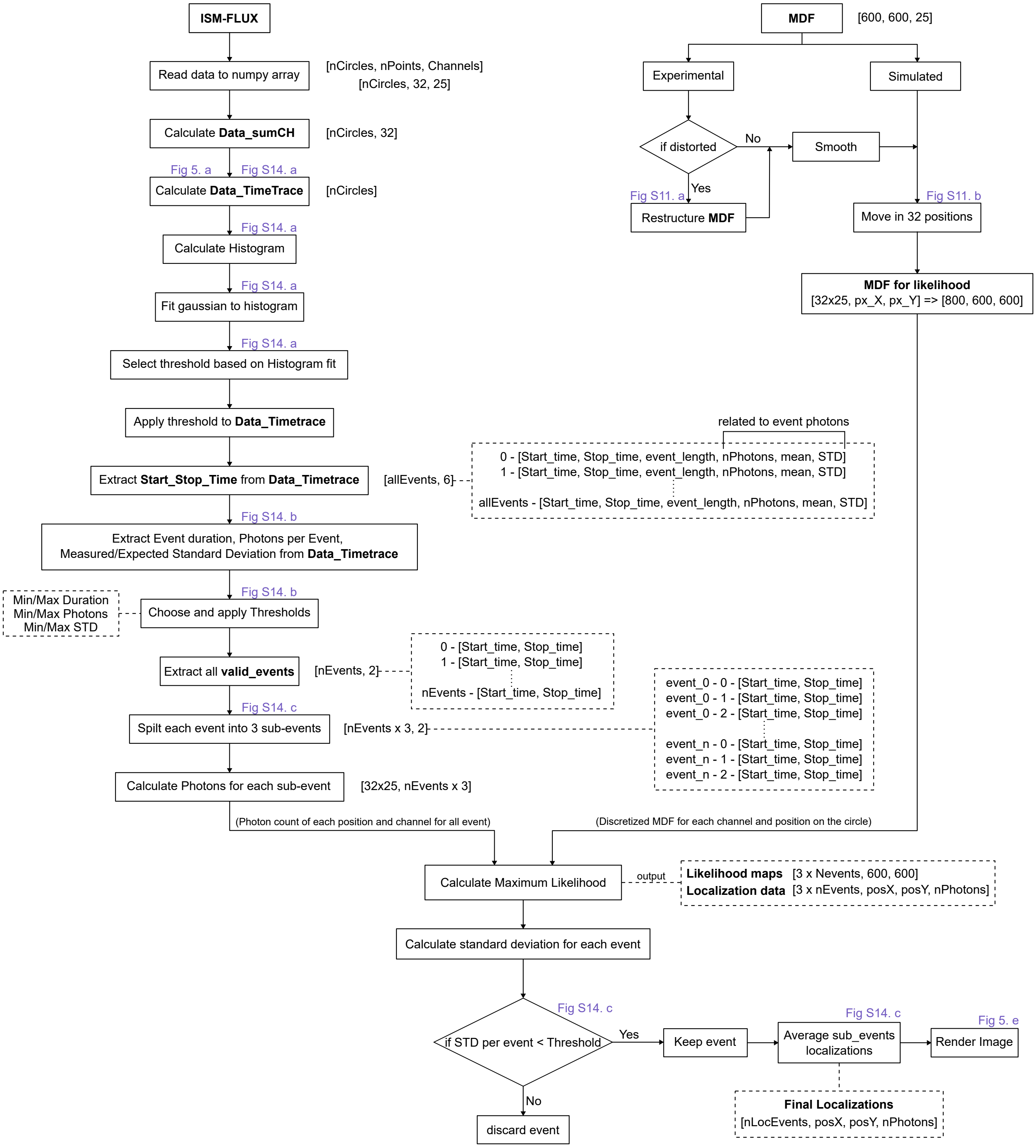

Fig. S16. Analysis pipeline for ISM-FLUX data analysis.
